# Hepatic infarction induced by HELLP syndrome: a case report and review of the literature

**DOI:** 10.1186/s12884-018-1799-9

**Published:** 2018-05-30

**Authors:** Qinyue Guo, Zhengfei Yang, Jian Guo, Lei Zhang, Lan Gao, Bo Zhou, Qindong Shi

**Affiliations:** 1 Department of Critical Care Medicine, the First Affiliated Hospital of Xi’an Jiaotong University, 277 Yanta West Road, Xi’an, 710061 Shaanxi China; 2 Sun Yat-sen Memorial Hospital, Sun Yat-sen University, Guangzhou, China; 3 Department of Hepatobiliary Surgery, the First Affiliated Hospital of Xi’an Jiaotong University, Xi’an, China; 4 Department of Respiratory Medicine, the First Affiliated Hospital of Xi’an Jiaotong University, Xi’an, China

**Keywords:** Preeclampsia, HELLP syndrome, Hepatic infarct

## Abstract

**Background:**

HELLP syndrome is a rare disease in China, and 20% of patients with severe preeclampsia have been accompanied with HELLP syndrome, which is characterized by the presence of hemolysis, elevated liver enzymes and low platelet count.

**Case presentation:**

In this case, we reported that a patient with preeclampsia was diagnosed with HELLP syndrome. Furthermore, hepatic infarction also was found via the computed tomographic (CT) images, which showed peripheral wedge-shaped inhomogeneous low attenuation in the right hepatic lobes via plain CT scan, and the low-density shadow and mottled appearance in the same areas where vessels were seen coursing through them via contrast-enhanced CT scan.

**Conclusions:**

Besides typical clinical manifestations of the pregnant patient with preeclampsia, the typical laboratory evidences were elevated liver enzymes and thrombocytopenia. The abdominal CT scan showed imaging features of hepatic infarction, which was helpful to identify the rare complication of HELLP syndrome. Thus, we diagnosed a patient with HELLP syndrome with hepatic infarction, though the patient had no chance to do the liver biopsy.

## Background

Preeclampsia (PE) is a pregnancy specific disorder which is characterized by new onset hypertension and proteinuria after the 20th weeks of gestation [1, 2]. In the most severe form of preeclampsia, the presence of HELLP syndrome (defined as: hemolysis, elevated liver enzymes and low platelet) with the addition of hepatic compromise [3], such as hepatic infarction or hematoma [4, 5], increased the risk of maternal morbidity and mortality [6].

HELLP syndrome should be carefully distinguished with the diseases, such as benign thrombocytopenia of pregnancy and virus hepatitis. It’s very important to identify the diagnosis of HELLP syndrome from other differential diagnosis. Once a clinical diagnosis of HELLP syndrome has been confirmed, it need aggressive interventions with control blood pressure, anti-seizure prophylaxis, corticosteroid treatment for fetal lung maturity enhancement, and expeditious delivery [7]. This case was collected in the First Affiliated Hospital of Xi’an Jiaotong University in China.

## Case presentation

A 31-year-old pregnant patient, with a history of two pregnancy losses at first half of pregnancy and no history of autoimmune or thromboembolic diseases, was evaluated at 33 + 4 weeks of gestation. An episode of symptomatic renal lithiasis requiring placement of double J stent at 3rd week of gestation was reported by the patient at admission.

The patient was admitted in local hospital due to dizziness, headache and blurred version,and worsening lower extremity edema 10 days ago. Examination results showed blood pressure 170/100 mmHg, proteinuria 3+ and occult blood 3+ by urinalysis. Immediate caesarean section was taken as diagnosed with severe preeclampsia. After the surgery, laboratory tests displayed elevated liver enzyme levels: peak aspartate transaminase (AST) and alanine transaminase (ALT) (1758 u/L and 2158 u/L respectively), decreased platelet count (20 × 10^9^/L) and hemoglobin value (65 g/L), increased white blood cells (WBC) (27.45 × 10^9^/L) and serum creatinine (233 umol/L) and urea (31.6 mmol/L). Symptomatic treatments were ineffective, so the patient was transferred to our hospital with the primary diagnosis of HELLP syndrome and severe preeclampsia.

On admission, the condition of the patient had worsened, and the heart rate was 120 beats per minute, the blood pressure was 138/74 mmHg (controlled by nitroprusside), and the temperature was 36.5 °C. The pitting ankle edema, headache and blurred version had gradually worsened accompanied with abdominal distension and weak chest, and decreased urine output. On physical examination, she appeared to be acutely ill with decreased breath sounds, the results of laboratory tests were not significantly improved than before, accompanied with negative urobilinogen, total bilirubin 25.5umol/L and reduced serum albumin (Table 1). The ultrasonic images of hepatic parenchyma were abnormal, which may result from blood supply deficiency, and massive hydrothorax and ascites. Based on all above, we diagnosed the patient as HELLP syndrome, but could not fully rule out other diseases with similar symptoms. The therapy including continuous renal replacement therapy (CRRT), plasma exchange and other supportive treatments such as red cell transfusion, platelet concentrates transfusions and antihypertensive were provided for the patient immediately.

**Table 1:** laboratory and blood chemical findings

	Local hospital	3th hospital day	17th hospital day
Glucose	Normal	Normal	Normal
Proteinuria	3+	3+	Normal
Aspartate transaminase (AST)(U/I)	1758	85	12
Alanine transaminase (ALT)(U/I)	2158	98	10
Total bilirubin (umol/L)	27.5	24	12
White blood cells (WBC)(*10^9^/L)	27.5	32.6	3.7
Hemoglobin (g/L)	65	65	81
Platelet count (*10^9^/L)	20	26	155
Serum creatinine (umol/L)	233	135	81
Urea (mmol/L)	31.6	11.6	3.6
Cholesterol(mmol/L)	4.09	2.99	3.05
Triglycerides(mmol/L)	3.31	2.01	1.70

**Fig. 1:**
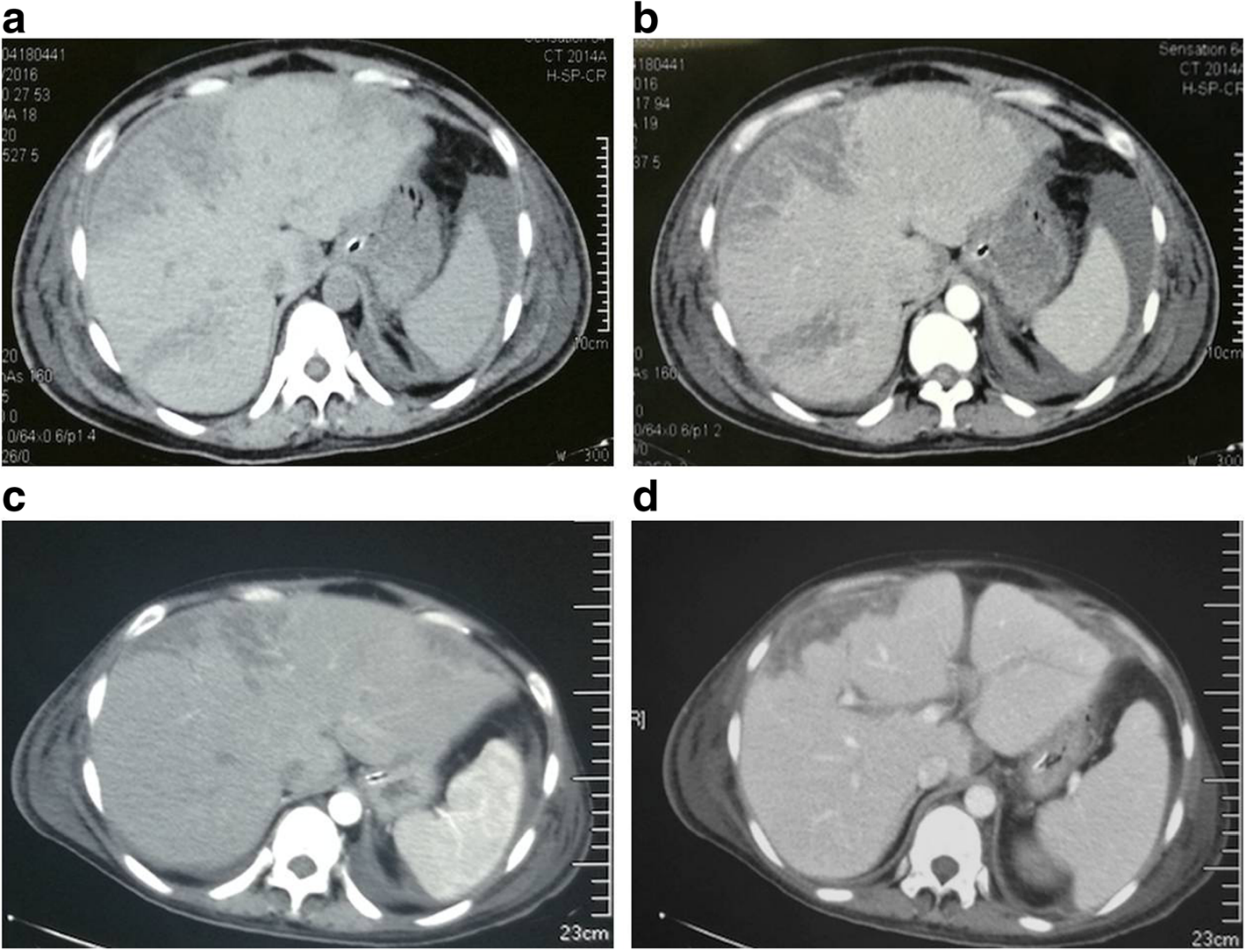
**a** Non-contras CT demonstrates peripheral wedge-shaped inhomogeneous low attenuation in the right hepatic lobes on 3th after admission. **b** Contrast-enhanced CT of the liver shows mottled appearance in the same areas where vessels were seen coursing through. **c** and **d** showed the damaged areas were reduced in the enhanced CT 17th days after admission

Three days after admission to our hospital, plain CT scan showed peripheral wedge-shaped inhomogeneous low attenuation in the right hepatic lobes (Fig. 1a), and the low-density shadow and mottled appearance in the same areas where vessels were seen coursing through them via contrast-enhanced CT scan (Fig. 1b). Base on CT images, liver infarction was diagnosed. Further tests showed that amylase in plasma and urine was negative, and hepatitis B antigens and antibody, and hepatitis A antibody were all negative, and the levels of glucose and blood ammonia were normal. Therefore, we further confirmed the diagnosis HELLP syndrome with liver infarction.

Then we continued treating the patient with antibiotics, CRRT, drainage of pleural effusion and ascites, and the supportive treatments. Intravenous dexamethasone 10 mg/d for 3 days was initiated. The results of laboratory tests were normalized within 17 days. The damaged areas were recovered on 17th days after admission via the second enhanced CT (Fig. 1c and d). She remained asymptomatic and had no complications during 6 months of follow-up.

## Discussion and conclusions

In this case, we reported that a pregnant woman with preeclampsia was diagnosed with HELLP syndrome (Hemolysis, increased liver enzyme, and low platelets count). Furthermore, hepatic infarction also found via the computed tomographic (CT) images, which showed peripheral wedge-shaped inhomogeneous areas of low attenuation in the right hepatic lobes via plain CT scan, and the low-density shadow and mottled appearance in the same areas where vessels were seen coursing through them via contrast-enhanced CT scan. Thus, the case of HELLP syndrome is rare to be found accompanied with liver infarction. Abdominal CT is often helpful for the management of HELLP syndrome to rule out hepatic hematoma, and in this case, we found that liver infarction was induced by HELLP syndrome.

Liver infarction is a very rare event because of hepatic distinct double blood supply. Most cases of liver infarction result from interference with arterial blood supply to the liver, due to pregnancy-induced hypertension (PIH), anti-phospholipid syndrome (APS), ischemic hepatitis, and portal vein thrombosis (PVT) and so on [8, 9]. Clinical manifestations of liver infarction are non-specificity, suddenly upper abdominal pain, fever, jaundice, and suddenly increased liver aminotransferases. The liver biopsy is helpful to distinguish liver infarction from other diseases such as liver abscess or cholangiocarcinoma, while, imaging examination also play an important role in diagnosis. Although hepatic infarction is nonspecific and may be caused by a variety of diseases, the CT images of the liver appear to be helpful for the differential diagnosis of liver dysfunction. A few cases of liver infarction induced by HELLP syndrome have been reported in China [10].

HELLP syndrome happened in about 20% of patients with severe preeclampsia [11]. The diagnosis of HELLP syndrome could be made through typical results of laboratory tests, including signs of hemolytic anemia and thrombocytopenia with platelets < 100,000 cells/ul, elevated AST, ALT and lactate dehydrogenase, associated with clinical manifestation of right upper quadrant pain, nausea, vomiting, malaise, headache and edema, but some women with HELLP syndrome may be asymptomatic.

In this case, proteinuria, pregnancy-induced hypertension, edema, elevated liver enzyme levels, decreased platelet count and hemoglobin, rapidly increased serum creatinine and urea all supported the criteria for both PE and HELLP syndrome [12], but the pathogenesis of both PE and HELLP is still unknown. Fortunately, CRRT and plasma exchange were effective for the patient, and after hormone therapy and supportive treatments, the damaged areas of liver were recovered and the patient got better.

Liver infarction is rare with the consequences of HELLP syndrome [13]. CT scan may be a useful method for the differential diagnosis, which showed peripherally wedge-shaped inhomogeneous areas of low attenuation with enhanced vessels coursing through these areas. Based on the clinical manifestation, the CT images, and other laboratory tests (For example the levels of glucose and blood ammonia are normal), we could rule out other diagnoses and make a definite diagnosis for HELLP syndrome with liver infarction.

In conclusion, we reported a patient diagnosed with the HELLP syndrome with hepatic infarction. The clinical manifestations of the pregnant patient with preeclampsia were headache, blurred version, abdominal distension and weak chest. The typical laboratory evidences were elevated liver enzymes, thrombocytopenia. Thus, we diagnosed the patient with the HELLP syndrome. The abdominal CT scan showed imaging features of hepatic infarction, which was helpful to identify the rare complication of HELLP syndrome.

## Abbreviations

AFLP: Acute fatty liver of pregnancy
ALT: Alanine transaminase
APS: Anti-phospholipid syndrome
AST: Aspartate transaminase
CRRT: Continuous renal replacement therapy
CT: Computed tomographic
DIC: Disseminated intravascular coagulation
PE: Preeclampsia
PIH: Pregnancy-induced hypertension
PVT,: Ischemic hepatitis, portal vein thrombosis
WBC: White blood cells
